# Editorial: Research advances of tuberculosis vaccine and its implication on COVID-19

**DOI:** 10.3389/fimmu.2023.1147704

**Published:** 2023-02-09

**Authors:** Wenping Gong, Jianping Xie, Hao Li, Ashok Aspatwar

**Affiliations:** ^1^ Tuberculosis Prevention and Control Key Laboratory/Beijing Key Laboratory of New Techniques of Tuberculosis Diagnosis and Treatment, Senior Department of Tuberculosis, The 8Medical Center of PLA General Hospital, Beijing, China; ^2^ Institute of Modern Biopharmaceuticals, State Key Laboratory Breeding Base of Eco-Environment and Bio-Resource of the Three Gorges Area, Key Laboratory of Eco-environments in Three Gorges Reservoir Region, Ministry of Education, School of Life Sciences, Southwest University, Chongqing, China; ^3^ College of Veterinary Medicine, China Agricultural University, Beijing, China; ^4^ Faculty of Medicine and Health Technology, Tampere University, Tampere, Finland

**Keywords:** *Mycobacterium tuberculosis*, vaccine, immune response, peptide, mechanism

## Introduction

Tuberculosis (TB) caused by *Mycobacterium tuberculosis* (*M. tuberculosis*) remains a global public health threat. As an aged disease, TB remains the leading cause of human death in 2021 from a single pathogen (https://www.who.int/news-room/fact-sheets/detail/tuberculosis). However, it is reported that the protective efficacy of BCG is highly variable and ranges between 0% and 80%, and its protection lasts only for 10 to 15 years (1). Therefore, developing a new effective TB vaccine is pressing and challenging for governments and scientists to end TB. Against this background, we launched a Research Topic in Frontiers in Immunology entitled “Research Advances of Tuberculosis Vaccine and its Implication on COVID-19”. This Research Topic published high-quality research articles that provide insight into the innate and adaptive immune responses during *M. tuberculosis* infection, trained immunity and protective efficacy induced by BCG or recombinant BCG (rBCG) vaccine, the potential role of the BCG vaccine on the prevention of COVID-19, and the latest progress of novel TB vaccines.

## BCG: Old tree sprouts new buds

Although BCG vaccination can effectively protect infants and young children from *M. tuberculosis* infection, especially in the prevention of severe TB diseases such as disseminated tuberculosis and tuberculous meningitis (TBM), its efficacy in adult pulmonary tuberculosis (PTB) is not satisfactory (2, 3). In recent years, with the rapid development of molecular biology, it has become possible to improve the efficacy of BCG by genetic modification. Recombinant BCG (rBCG) strains are developed by expressing foreign antigens, human cytokines, and pro-apoptotic factors. In this Research Topic, two studies developed new rBCG strains and investigated their efficacy in animal models. Based on previous findings that a recombinant BCG expressing the LTAK63 adjuvant (rBCG-LTAK63) can protect mice from *M. tuberculosis* infection based on increased early and long-term immune responses (4, 5), Trentini et al. evaluated the effect of rBCG-LTAK63 in the treatment of mice infected by *M. tuberculosis*. They found that both the bacterial burden and the area of inflammation in the lungs of mice treated with rBCG-LTAK63 were less than those of the BCG control group. Similarly, Ning et al. further evaluated the protection efficiency of the rBCG-DisA vaccine in mice infected with *M. tuberculosis*. Their data demonstrated that the rBCG-DisA vaccine induced robust trained and adaptive immunities and showed an excellent performance in the prime-boost strategy against *M. tuberculosis* infection in mice. Currently, three rBCG vaccines (VPM1002, rBCG30, and AERAS-422) have been evaluated in clinical trials, but the rBCG30 and AERAS-422 trials were stopped due to safety concerns (6, 7). Therefore, attention must be paid to the safety of the rBCG vaccine when designing a novel rBCG strain.

In addition to improving the BCG strains by genetic engineering, BCG’s prime-boost strategy is also a rational way to develop new TB vaccines. Lv et al. primed C57BL/6 mice with BCG followed by two or three booster immunizations with MH (Mtb10.4-HspX) or EC (ESAT6-CFP10) subunit vaccine. The results showed that two booster doses of MH at 12 and 24 weeks or three booster doses of EC at 12, 16, and 24 weeks after BCG primary immunization could induce significantly high levels of long-term memory T cells and improve the protective efficiency of the subunit vaccines. Negi et al. reviewed progress in BCG vaccination programs, including BCG vaccination in children and potential strategies to enhance BCG-induced protection in adults.

## BCG and COVID-19: More evidence is needed

Trained immunity is defined as the ability of innate immune cells to generate heterologous memory in response to specific exogenous exposures (8). Previous ecological, analytical, and animal studies demonstrate that the trained immunity induced by the BCG vaccine might protect against infections of various respiratory pathogens other than *M. tuberculosis*, including SARS-CoV-2 (9). In this Research Topic, Anjos et al. conducted a randomized Phase II clinical trial to evaluate the efficacy and safety of BCG to prevent SARS-CoV-2 infection in health care workers. They found that revaccination with BCG produced using Moscow strain was safe and resulted in a lower, but not statistically significant, incidence of COVID-19 positivity. Furthermore, Gong et al. published an opinion article to discuss the natural effect of BCG vaccination on COVID-19. They discussed the non-specific immune response induced by the BCG, analyzed early findings from the ecological and analytical studies, and pointed out that the heterogeneity of these findings might originate from some confounding factors. In addition to exploring the potential roles of BCG vaccine in the prevention and controlling of COVID-19, a study has evaluated the role of a TLR2/6 agonist, Pam2CSK4, in COVID-19 vaccine (Qiao et al.). Qiao et al. used Pam2CSK4 to enhance the immune responses induced by a nanoparticle vaccine against COVID-19, they found that with the help of TLR2/6 agonist Pam2CSK4, the nanoparticle vaccine could induce a significantly higher levels of antigen-specific neutralizing antibodies and Th1-biased immune response by upregulating genes involved in the migration, activation, and proliferation of leukocytes. These results suggest that Pam2CSK4 might be a promising adjuvant for the nanoparticle vaccine, which highlights a new tool to enhance the immunogenicity of TB vaccines.

## New TB vaccines: A potential new force to end TB

The growing evidence indicate that humoral immune responses play important roles against *M. tuberculosis* infection, which suggests that the humoral responses as well as the cellular immune responses elicited by the vaccines shall be factored into new vaccines design (3, 10–12). Wu et al. found a secreted Rv1579c (EST12) protein from the *M. tuberculosis* region of deletion 3 (RD3) could promote Myc binding to the promoters of IL-6 and TNF-α cytokines, nitric oxide (NO), and inducible nitric oxide synthase (iNOS) *via* activating JNK-AP1-Myc signaling pathway, which facilitates host clearance of *M. tuberculosis*. This study provides new insight into the mechanism of interaction between *M. tuberculosis* and the host, as well as potential target for developing new TB vaccines.

A viral vector is a newly discovered vaccine vector that relies on molecular biology techniques to integrate the gene of the target antigen of *M. tuberculosis* into its genome and deliver it to the host cell for expression of the target antigen to induce an immune response (13). Hu et al. reviewed the research progress of virus-vectored TB vaccines, especially those that have been in clinical trials and those that are still in preclinical research stage but most promising, they also provided an update on the latest tools and concepts that facilitate TB vaccine research and development.

Furthermore, DNA vaccines against TB have been investigated in this Research Topic. Liang et al. constructed an *M. tuberculosis ag85a/b* chimeric DNA vaccine and evaluated and compared its immunotherapeutic effects under different immunization doses and routes. They demonstrated that the *ag85a/b* chimeric DNA vaccine killed *M. tuberculosis* and eliminated by inducing a Th1-type cellular immune response. Weng et al. constructed four DNA vaccines (A39, B37, B31, and B21) and found that the B21 DNA vaccine could induce effector memory and central memory T cells and produce the strongest Th1/Th17 and CD8^+^ cytotoxic T lymphocyte responses to reduce mycobacterial loads in the mice with latent *M. tuberculosis* infection (LTBI), suggesting that the B21 DNA vaccine can enhance T cell responses and control the LTBI infection.

Different from the above vaccines, peptide-based TB vaccines have emerged based on the rapidly developing bioinformatics and immunoinformatics technologies in recent years. Gong et al. provided the first detailed and comprehensive review of the peptide-based TB vaccine. They summarized the development of bioinformatics tools used in the identification of potential antigens of *M. tuberculosis*, prediction of T cell and B cell epitopes, analysis of epitope immunogenicity, antigenicity, allergenicity, and toxicity, and construction of peptide-based vaccines.

## Where to go: Challenges and opportunities

Since the only TB vaccine, BCG, was developed more than a hundred years ago, no new TB vaccine has been approved for use to prevent and control TB. Although 14 TB vaccines have entered the clinical trial stage, the final protection efficiency of the highly anticipated M72/AS01_E_ vaccine after 36 months of follow-up is only 49.7% (14), which makes the research of TB vaccine enter the darkest moment. A better understanding of the pathogenic mechanism of *M. tuberculosis* and the host’s immune protection mechanism would help solve the current stagnation of TB vaccine development. To overcome these difficulties, future research should focus on the following aspects: 1) Novel discovery and theory on the pathogenic mechanism of *M. tuberculosis* and the immune protection mechanism of the host; 2) The trained immunity induced by BCG vaccine and the rBCG vaccine development; 3) Protective immune responses induced by the immunotherapeutic vaccines; 4) Novel TB vaccines and their protective mechanisms in pre-clinical and clinical studies, especially the vaccines based on new technologies or antigens; 5) New technologies such as bioinformatics, immunoinformatics, and reverse vaccinology in the design and construction of novel TB vaccines; 6) Animal models that more accurately predict the heterogeneity of the immune response against *M. tuberculosis* in humans; 7) Vaccine immunization strategies, such as adjuvants, immunization routes, doses, and times; 8) Therapeutic vaccines to prevent relapse following cure or adjunctive treatment of TB and passive immunization such as antibodies directed against *M. tuberculosis* antigens.

In summary, the collection of papers published in this Research Topic (Volume 1) demonstrates the current status of the research and development in the areas of TB vaccines. In the second volume, we will continue to focus on the above eight future research directions, showcase the achievements of TB vaccine research and the latest immunization strategies from different perspectives, and contribute to the vision of ending TB by 2035 formulated by the WHO.

## Author contributions

All authors listed have made a substantial, direct, and intellectual contribution to the work and approved it for publication.
